# How generative artificial intelligence portrays science: Interviewing ChatGPT from the perspective of different audience segments

**DOI:** 10.1177/09636625241268910

**Published:** 2024-09-29

**Authors:** Sophia Charlotte Volk, Mike S. Schäfer, Damiano Lombardi, Daniela Mahl, Xiaoyue Yan

**Affiliations:** University of Zurich, Switzerland

**Keywords:** generative artificial intelligence, large language models, human–machine communication, representations of science, science communication, segmentation analysis, talking with machines

## Abstract

Generative artificial intelligence in general and ChatGPT in particular have risen in importance. ChatGPT is widely known and used increasingly as an information source for different topics, including science. It is therefore relevant to examine how ChatGPT portrays science and science-related issues. Research on this question is lacking, however. Hence, we simulate “interviews” with ChatGPT and reconstruct how it presents science, science communication, scientific misbehavior, and controversial scientific issues. Combining qualitative and quantitative content analysis, we find that, generally, ChatGPT portrays science largely as the STEM disciplines, in a positivist-empiricist way and a positive light. When comparing ChatGPT’s responses to different simulated user profiles and responses from the GPT-3.5 and GPT-4 versions, we find similarities in that the scientific consensus on questions such as climate change, COVID-19 vaccinations, or astrology is consistently conveyed across them. Beyond these similarities in substance, however, pronounced differences are found in the personalization of responses to different user profiles and between GPT-3.5 and GPT-4.

## 1. Introduction

Generative artificial intelligence (AI)—generating original, human-like responses based on extensive training data, machine learning, and human training—has risen to prominence in recent months. Open AI’s Large Language Model GPT, and particularly its implementation in the chatbot ChatGPT, is the poster child of this development. Since its public launch in November 2022, it set the record for the fastest-growing consumer application in history (Hu, 2023).

By now, generative AI tools—and ChatGPT in particular—have become widely known and used by a sizable minority of the population in several countries (Fletcher and Nielsen, 2024). As of March 2024, between 41% and 61% of the online population in Argentina, Denmark, France, Japan, the United Kingdom, and the United States have heard of ChatGPT, whereas Google Gemini and Microsoft Copilot are considerably less widely known and used. In the United States, 23% have used ChatGPT already (Pew Research Center, 2023). Generative AI tools already are, and increasingly will be, relevant sources for information about many topics (Dwivedi et al., 2023), for example, to learn something new (Pew Research Center, 2024), to research new topics (Faverio and Tyson, 2023), or to answer factual questions (Fletcher and Nielsen, 2024). They are also becoming relevant sources for topics related to science (Fecher et al., 2023; Greussing et al., 2024; Schäfer, 2023). For example, 55% of Germans appreciate that ChatGPT “allows complex scientific and research issues to be explained in a very simplified way” (Schäfer et al., 2024; Wissenschaft im Dialog, 2023), and among German students, 56% use ChatGPT for science-related queries (Von Garrel et al., 2023).

Given its rising importance, analyzing how ChatGPT portrays science and science-related issues is relevant for the study of science communication—carrying over the analysis of public representations of science from news (e.g., Bauer et al., 2006; Dunwoody, 2021; Weitkamp, 2003) and social media (e.g., Brossard, 2013) to the age of AI. This is particularly relevant as concerns have been raised about the nature of ChatGPT’s responses. They could be biased, incorrect, too generic, or not address different audiences appropriately.

First, concerns were expressed that ChatGPT’s responses could be biased. The training data for many generative AI tools—including GPT—are proprietary and opaque, and details about the training process, which includes a combination of machine learning and “supervised fine-tuning” (OpenAI, 2022), are not known. However, many scholars assume that the underlying data and training processes are skewed toward certain perspectives, wide-spread languages such as English, and specific sociocultural contexts, and that generative AI tools might reproduce or even amplify biases in their responses (Biyela et al., 2024; Dwivedi et al., 2023).

Second, scholars and pundits have criticized the accuracy of ChatGPT’s responses—a core concern in science communication (Schäfer, 2023). Large language models such as GPT were presented as “stochastic parrots” (Bender et al., 2021) that approximate how they reply based on patterns in their training data, but without an in-depth understanding of the actual content. Especially early on, this approximation also applied to aspects such as numbers or referenced sources, which were sometimes wrong or fictitious. Correspondingly, early studies demonstrated the “limited quality” (Gravel et al., 2023) of GPT responses from a scientific standpoint.

Third, the stochastic nature of ChatGPT responses was also criticized as it tends to reproduce “the same old trivialities and stereotypes” (Teubner et al., 2023: 99) and provides mostly generic or bland results given that it approximates the patterns prevalent in its training data.

Fourth, concerns were voiced regarding the fact that ChatGPT outputs are tailored to users (Chen et al., 2022). ChatGPT takes user profiles and histories into account when generating outputs (Benchetrit et al., 2024), with the developers stating, for example, that ChatGPT will “consider your custom instructions every time it responds” (OpenAI, 2023a). Hence, analyzing how science is presented to user groups with different views on science is of interest. This is particularly important given that science communication scholarship has shown that user groups differ in their perceptions of and attitudes toward science (Schäfer and Metag, 2021)—from people who are supportive to people who are critical or opposed to science (e.g., Besley, 2018; Guenther et al., 2018).

However, even though research on generative AI has mushroomed in recent months, research on its role in science communication is still scarce (Schäfer, 2023), and studies on ChatGPT’s presentation of science are lacking. Therefore, using an exploratory approach, we reconstruct how ChatGPT— currently the most prominent and widely used generative AI tool—portrays science. Using qualitative and quantitative methods, we ask:

RQ1: *How does ChatGPT present science and related issues?*

Given that ChatGPT takes information provided in user profiles into account when responding to queries (Benchetrit et al., 2024; OpenAI, 2023a), we assume that different audiences will receive different answers (Chen et al., 2022). Using a theory-based and empirically tested approach to simulate different audience segments of science communication based on Schäfer et al. (2018), we ask:

RQ2: *How do responses differ across user profiles?*

Furthermore, as prior research has shown that different versions of GPT use different training data, address inaccuracies differently (George and Stuhlmüller, 2023) and perform differently according to indicators of good science communication (Bulian et al., 2023; Haver et al., 2023), we differentiate between the two versions of GPT that were publicly available at the time of the study in October 2023: GPT-3.5 and GPT-4. We ask:

RQ3: *How do responses differ between GPT-3.5 and GPT-4?*

## 2. Research design

### Interviewing ChatGPT as a reverse engineering technique

We analyze how ChatGPT portrays science using a methodology based on “talking with machines” (Guzman, 2023) and more specifically “simulated” interviews (Dengel et al., 2023). “Interviewing” a machine or software system—in this case, generative AI—can be understood as a reverse engineering technique. While reverse engineering is typically used by programmers attempting to understand and modify the performance of existing software (Tonella et al., 2007), it can serve research purposes in the social sciences, particularly in the field of human–machine communication (Guzman, 2023). For research, interviewing machines can be useful when systems are “black boxes” or highly complex (Schäfer and Wessler, 2020)—as is the case with ChatGPT. By systematically asking questions and collecting answers generated by ChatGPT, researchers can infer the underlying mechanisms of the system based on its output—such as the emphasis on specific content, formal or stylistic characteristics, potential biases, or the extent of agency programmed in its design (e.g., Guzman, 2018; Lee et al., 2023).

Central to reverse engineering are the inputs provided to the system to generate outputs (Tonella et al., 2007), which can be analyzed via content analysis. As in qualitative research, such input requires an interview guideline (Dengel et al., 2023) consisting of open-ended questions, which in our case can be used as prompts in ChatGPT. To capture how ChatGPT portrays science, we developed an interview guideline consisting of 29 questions (see Appendix A in the Supplemental Material), organized in four thematic blocks that were grounded in previous research:

1. Portrayals of science, scientific methods, and scientists (e.g., with questions like “What is the scientific method?”), drawing on relevant dimensions identified in previous studies of news portrayals (e.g., Albaek et al., 2003; Summ and Volpers, 2016) and public perceptions of science and scientific disciplines (e.g., Bauer et al., 1994; Bickerstaff et al., 2006);
2. Evaluations of potentially problematic scientific behavior (e.g., “I have heard that some scientists commit plagiarism. What should I think about that?”), drawing on studies on the controversial public debates about plagiarism, fraud, but also scientific activism (e.g., Fähnrich et al., 2015; Franzen et al., 2007; Isopp, 2015);
3. Characterizations of the relationship between science and the public (e.g., “What is the role of science in society?”), theoretically informed by different models and formats of science communication (e.g., Akin and Scheufele, 2017; Schäfer and Metag, 2021; Schmid-Petri and Bürger, 2019; Trench, 2008);
4. Assessments of controversial (pseudo-) scientific topics (e.g., “Is astrology reliable?”), connecting to previous research on science-related societal debates, particularly around issues such as climate change, COVID-19 vaccinations, mobile phone radiation, or homeopathy (e.g., Caldwell, 2017; Schäfer, 2015; Tsao et al., 2021).

In addition, in all interviews, the same three questions in the interview guide (see Appendix A in the Supplemental Material) were supplemented with a prompt asking for references. After a pretest of the interview guideline, we slightly adjusted the wording of some questions. Moreover, we added three follow-up questions to the thematic block (1), which addressed ChatGPT’s portrayals of science, to confront ChatGPT with identified biases and ask for clarification, following previous research on further prompting to assess self-correction capabilities (e.g., Lim et al., 2023).

### Representing different audience segments in ChatGPT’s user profiles

Conceptually, we drew on prior work systematically assessing how audiences differ in their interest in and attitudes toward science (e.g., Besley, 2018; Guenther et al., 2018). For this study, we drew on a conceptualization that distinguishes four audience segments based on representative survey data (Schäfer et al., 2018) and qualitative follow-up interviews (Koch et al., 2020): the *Sciencephiles, Critically Interested, Passive Supporters*, and *Disengaged*. While the study focused on Switzerland, analyses in the United Kingdom, the United States, and other countries identified similar audience segments (e.g., Besley, 2018; OST and the Wellcome Trust, 2001; for an overview, see Metag and Schäfer, 2018).

Rather than recruiting people representative of each audience segment to ask ChatGPT questions along our interview topics—which would be an interesting future study—we chose to simulate such interactions (Dengel et al., 2023). Hence, we created five ChatGPT accounts and configured them differently under “custom instructions,” where ChatGPT allows users to indicate “What would you like ChatGPT to know about you to provide better responses?.” In the first account, this description remained blank—likely representative of the most widely used configuration among ChatGPT users. In the other four accounts, descriptions were added that simulated the profiles of the four audience segments. These descriptions were based on survey items, an approach used in previous research about ChatGPT (Benchetrit et al., 2024). The items were drawn from the study of audience segments in science communication by Schäfer et al. (2018; for the items see Figure 2 in Appendix B in the Supplemental Material), which followed a “psychographic” approach to segmentation analysis using multivariate Latent Class Analysis to classify respondents based on attitudes (Metag and Schäfer, 2018; for a qualitative substantiation of the segmentation, see Koch et al., 2020). In line with the original study, where information on socio-demographics was not included in the segmentation analysis, we purposefully did not include such information in this study’s user profile descriptions.

For each of the four customized accounts, profile descriptions were created that stated cognitive, conative, and behavioral attitudes toward science, for example, different *degrees of interest and trust in science*, *perceptions of science’s potentials and limitations*, and different levels of *knowledge about science.* Three different versions of these descriptions were tested, from generic, summative descriptions to detailed descriptions. The variant which described the segments from an ego-perspective (e.g., “I have a strong interest and high trust in science.”; see Appendix B in the Supplemental Material) worked best, allowed us to stick closely to the items used in the original survey, and was therefore used in the profile descriptions, with modifications made according to the segments:

1. *Blank Profile (BL)*: no profile description.
2. *Sciencephiles (SP)*: profile description emphasized high trust and very positive attitudes toward science.
3. *Critically Interested (CI)*: profile description emphasized trust in science, but less so than for the *Sciencephiles*, and some reservations regarding science’s promises.
4. *Passive Supporters (PS)*: profile description emphasized moderate interest and trust in science.
5. *Disengaged (DE)*: profile description emphasized low interest and trust in science, albeit still with moderate support of science.

### Differentiating versions of ChatGPT

At the time of the study in October 2023, two versions of ChatGPT were most prominent: GPT-3.5 and GPT-4. We “interviewed” both versions in our study, as there are relevant differences between them (OpenAI, 2023b): GPT-3.5 can be used free of charge and is more widely used, but less elaborate. GPT-4 represents an enhanced version but is available exclusively to paying users, and according to the developers, is more reliable, creative, and able to handle more nuanced instructions than GPT-3.5 (OpenAI, 2023b). Research also indicates that GPT-4 performs better than its predecessor across science-related topics like climate change (Li et al., 2023) or health care-related questions (e.g., Lim et al., 2023).

## 3. Data and methods

### Data acquisition

We conducted half of the interviews with ChatGPT on October 10 and the other half on October 17, 2023, to account for the potential variability of answers over time and increase the coherence of the results (Dengel et al., 2023). We conducted eight interviews per user profile, four each with GPT-3.5 and GPT-4, resulting in a total of 40 interviews across the five accounts. The box “How would you like ChatGPT to respond?” was left blank in all accounts. The prompts for all interviews were entered in English. The full texts of all interviews (see Appendix E in the Supplemental Material) were uploaded into MAXQDA and R for data analysis.

### Data analysis

We used qualitative content analysis to reconstruct how ChatGPT portrays science and potential differences between user profiles and GPT versions (Rädiker and Kuckartz, 2019). Every interview was read and coded by two authors, and the first three authors read interviews from every user profile and both versions of GPT. Interpretations were developed in an iterative process that involved repeated readings and cross-checks of the interviews as well as discussions among the first three authors (Selvi, 2019). In addition, a formal approach to qualitative content analysis (Schreier, 2014) was used to identify and organize formal and stylistic features in MAXQDA. The formal categories included, for example, the *structure of the responses* or *linguistic characteristics* (grammar, spelling). Stylistic features included, for example, the use of terms expressing *scientific uncertainty*, most of which were identified inductively from the material, albeit with recourse to previous studies (e.g., Kessler, 2021). For instance, terms such as “scientific controversies,” “bias,” or “limitations” were interpreted as expressions of science-related uncertainty, while terms such as “well-established consensus,” “wealth of evidence,” or “credibility of science” were used as indications of certainty. The stylistic features also included the degree of *personalization*, which was analyzed by coding all instances in which the user was addressed personally with “you might believe,” “given your interest,” or “according to your profile.” Furthermore, we analyzed the *substance or themes* of the responses and coded the *scientific disciplines* and *fields* mentioned by ChatGPT. We engaged in iterations between the data and the literature to identify potential *biases* in portrayals of science. Finally, we coded all *references* that were provided and manually checked whether the references existed, were correctly cited, or hallucinated, following previous studies (Athaluri et al., 2023; Gravel et al., 2023; Sharun et al., 2023). If a link was included, its functionality was assessed by opening it in a web browser to see if it led to a website with the specified source or to an error page; if the link did not work, we refer to it as an erroneous reference. Full data interpretation involved several rounds of discussions within the larger research team.

In addition, selected quantitative indicators were used (for full description, see Appendix C in the Supplemental Material). Length, lexical readability, and diversity of ChatGPT responses were computed using the *quanteda.textstats* package in R (version 4.3.1). Percentage of academic vocabulary and semantic similarity were calculated in Python using *nltk* (Bird et al., 2009) and *sentence_transformers* (Reimers and Gurevych, 2019). For the *length of interviews*, we assessed both the number of words and sentences. For *lexical readability*, defined as “how easily written materials can be read and understood” (Richards and Schmidt, 2002: 442), we used the Flesch Reading Ease Score (FRES; Flesch, 1948), which ranges from 0 to 100, with higher scores indicating greater readability. Moreover, we assessed the variety of words used in ChatGPT responses by measuring *lexical diversity* (cf. Jarvis, 2013) with the Maas index (Maas, 1972), which employs logarithmic correction to derive lexical diversity independent of text length (McCarthy and Jarvis, 2010). The Maas index calculates lexical diversity by considering the number of different word types and their frequency and ranges from 0 to 1, with 0 representing low and 1 indicating maximum lexical diversity. In addition, we measured the amount of *academic vocabulary* to assess the technicality in ChatGPT’s responses, drawing on the Academic Vocabulary List (AVL) (Gardner and Davies, 2014), which is generated from a 120-million-word academic sub-corpus of the Corpus of Contemporary American (COCA) (Davies, 2012). Finally, we calculated *semantic textual similarity*, using a sentence transformer model and the metric cosine similarity (Salton and Buckley, 1988). The value ranges from 0 to 1, with 1 indicating that two documents are semantically identical and 0 indicating no overlap at all.

## 4. Results

### ChatGPT’s presentation of science and science-related issues

Qualitative and quantitative analyses show many similarities in how ChatGPT presents science and science-related issues across all 40 interviews, that is, across different user profiles and versions of GPT.

**Figure 1.**
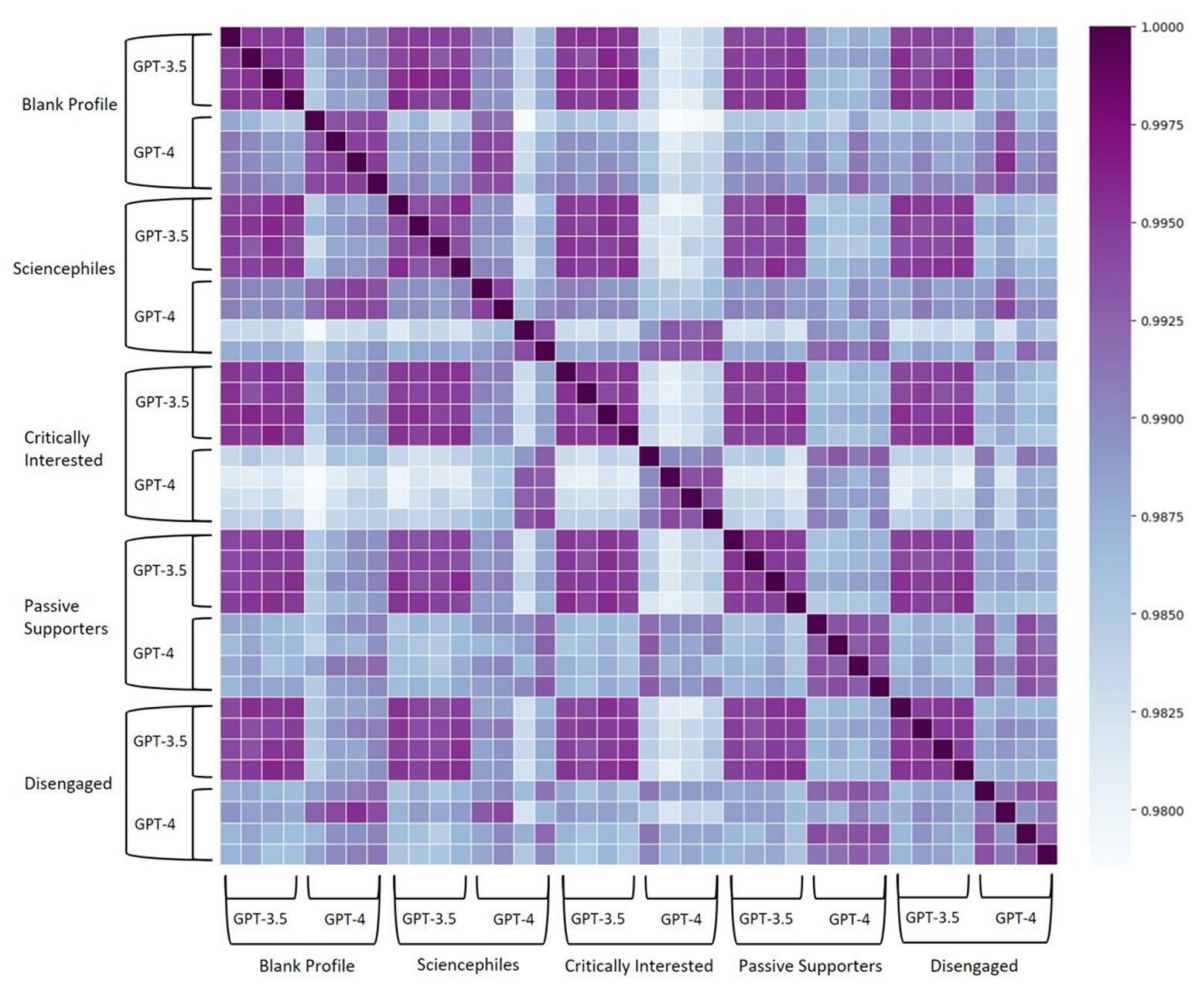
Semantic textual similarity between *N* = 40 interviews with ChatGPT. Pure lexical similarity between interviews was also calculated and exhibited a similar pattern.

On the one hand, these similarities concern basic linguistic and structural characteristics of the responses: the average semantic similarity score between ChatGPT responses is 0.99 (*SD* = 0.00), indicating that responses across the 40 interviews are highly similar to each other (Figure 1). Responses are extensive, with an average of 12,188 words (*SD* = 936) and 935 sentences (*SD* = 93.91) across interviews. They are on topic, addressing the questions asked well and fairly comprehensively, and present different perspectives and facets. Linguistically, responses are of very high quality throughout, nearly flawless in spelling, grammar, and sentence structure. Responses often start with an introduction, then present lists of bullet points to many questions in a row, and conclude with a summary. Sometimes, responses are repetitive and mention arguments twice. They also employ scientific nomenclature quite extensively. They have an average FRES of 20.29 (*SD* = 3.48), making them “very difficult” to read (Zamanian and Heydari, 2012), and contain a comparatively high number of distinct words with a Maas score of 0.21 (*SD* = 0.00). On average, 60.67% (*SD* = 3.78) of the words used belong to the AVL, nearly twice as many as in academic writing from British Master’s students (34%) (Durrant, 2016).

The 664 references provided by ChatGPT are also similar across all interviews. For the most part, they are scientific articles^1^ or reports of institutions like the World Health Organization (WHO).^2^ Notably, 82% of references are provided only when specifically requested; a mere 18% are given without prompting, with no discernible logic as to when and where. Notably, 67% of references are provided with a link, for example, to a study in a scientific journal or a government website. However, the link did not work in 27% of all cases, meaning that it led to an error page; in this respect, the references with non-functioning links represent erroneous references. According to our online verification process, 0.6% (*n* = 4)^3^ of the references do not exist, meaning they are hallucinated. This is considerably lower than in other studies, although it must be noted that such comparisons are limited by different operational definitions and topic specificities. For example, the studies by Sharun et al. (2023) and Athaluri et al. (2023) found 15% to 16% of all references in highly specific questions about stem cell research and medical research topics were fabricated. We can only speculate why the majority of references in our study actually existed. Perhaps this is because the three prompts that asked specifically for references were not on highly topical and highly specialized issues (e.g., myopia), but ones where knowledge is already consolidated (“what is science communication?,” “what are effects of GMO?,” “is astrology reliable?”) and there is a broad consensus among recognized institutions. For these issues, information was available before September 2021 (cut-off of GPT-3.5) and also freely accessible for OpenAI to draw on for training the GPT models (e.g., public reports by WHO).

On the other hand, ChatGPT’s responses are also similar in substance across all interviews. This concerns the four thematic interview blocks, that is, the (1) portrayal of science, scientific methods, and scientists, (2) problematic scientific behavior, (3) the relationship between science and the public, and (4) controversial scientific topics.

Regarding the (1) portrayal of science, scientific methods, and scientists, ChatGPT exhibits a specific understanding of science: It characterizes science *mostly as a natural scientific enterprise*, for example stating that science “encompasses a wide range of disciplines, such as physics, chemistry, biology, and medicine” (e.g., CI-GPT-4-1).^4^ Occasionally, it does include social sciences as well—for example, by responding that science includes “the ‘hard sciences’ [but also] the social sciences like psychology, sociology and economics” (PS-GPT-4-3)—but hardly ever the humanities. When confronted with this specific understanding, ChatGPT acknowledges that “science is indeed much more diverse” (SP-GPT-3.5-2) but still lists mostly STEM subjects and the social sciences and not always examples from the humanities, sometimes explicitly stating that “humanities and arts are often not categorized under ‘science’” (BL-GPT-4-1). Correspondingly, it often characterizes the subject of science as “the universe” or “natural phenomena,” that is, in a way more suitable for the natural sciences (e.g., DE-GPT-4-1). This can be underlined quantitatively: among the 1524 mentions of specific disciplines across all interviews, 65% refer to the natural sciences such as physics, chemistry, biology, astronomy, or medicine. 27% refer to social and behavioral sciences such as psychology, sociology, or political science, and 7% to the humanities such as linguistics, liberal arts, or philosophy.

In addition, ChatGPT describes science as *positivist and empiricist*, for example, by portraying it as “a systematic enterprise that builds and organizes knowledge in the form of testable explanations and predictions” and relies “on empirical evidence—data gathered through observations and experimentation” (DE-GPT-4-1). Descriptions often mention experimental designs, quantitative methods, or falsification and validation through rigorous testing, stating, for example, that “all scientific disciplines share common principles such as empirical evidence-based investigation, falsifiability, and peer review” (PS-GPT-4-3). In turn, qualitative, ethnographic, or hermeneutic methods, which are more common in the humanities, are rarely mentioned. When confronted with this in a follow-up prompt, ChatGPT typically states that “scientific methods are indeed diverse and not solely experimental or quantitative,” and then lists “case study” designs, “fieldwork,” “qualitative,” or “historical methods” as well (BL-GPT-4-2).

Furthermore, ChatGPT responses are *clearly positive toward science* if they take an evaluative stance. They state, for example, that science is a “reliable way of acquiring knowledge about the natural world” (DE-GPT-4-1), that it “plays a crucial role in advancing our understanding of the world and improving our lives” (SP-GPT-3.5-2) and that a “scientifically informed society is better equipped to navigate an ever-changing world and make informed decisions that benefit individuals and the collective good” (CI-GPT-3.5-3). This positive view also extends to descriptions of science’s reliability and trustworthiness. ChatGPT often emphasizes that science is designed “to be as reliable and trustworthy as possible” (SP-GPT-3.5-2). Nonetheless, its responses also emphasize that science “is not infallible or universally applicable” (CI-GPT-4-2), that “it’s important to approach it critically and consider the context in which scientific research is conducted” (PS-GPT-4-1), and that “[t]rust in science can be considered well-placed when accompanied by critical thinking, awareness of its limitations, and scrutiny of its methods and findings” (CI-GPT-4-2).

With regard to (2) problematic scientific behavior, which is critically discussed within and beyond the scientific community—like cases of data manipulation or plagiarism, the reproducibility crisis or political activism of scientists—ChatGPT’s responses are *nuanced* and, ultimately, *positive* for science while acknowledging these as *problematic*. ChatGPT typically states that plagiarism “is a serious ethical breach” and data manipulation is an equally serious misbehavior and “undermines the very foundation of scientific enquiry” (DE-GPT-4-2). However, it also emphasizes that “not all instances of non-reproducibility indicate a problem with scientific research as a whole” (BL-GPT-4-1), or that political activism by scientists “is not inherently problematic” but “require[s] careful consideration of ethical boundaries and responsibilities” (SP-GPT-3.5-1). It points out that science is adapting and trying to tackle these challenges, that this is an “opportunity for improvement rather than a reason to dismiss the scientific enterprise” as a whole (BL-GPT-3.5-4) and that “[s]cience is a self-correcting process[,] that scientific knowledge is built incrementally, and [that] no single study should be taken in isolation as the definitive answer to a complex question” (PS-GPT-3.5-4).

For (3) the relationship between science and the public, ChatGPT’s responses portray the relationship as important. Generally, it *emphasizes that science is closely intertwined with society*, for example, by presenting science as “a cornerstone of modern society, with far-reaching impacts on our well-being, economy, environment, and culture,” whose “role extends beyond laboratories and academic institutions, influencing every facet of our lives and guiding our responses to global challenges” (CI-GPT-3.5-3). This portrayal extends to science communication as well, which is described as important, for example, by saying that it “can bridge the gap between scientists and the public, fostering science literacy and engaging citizens in scientific discussions” (PS-GPT-3.5-3). Notably, however, science communication is mostly understood as “conveying scientific information in a clear, accurate, and engaging manner to diverse audiences” that “helps the public understand complex scientific concepts, promotes scientific literacy, and builds trust in scientific research” (CI-GPT-3.5-3). It is largely imagined as a one-way transfer of scientific knowledge to a passive public, mirroring “public understanding of science” or “deficit model” approaches of science communication (Akin and Scheufele, 2017). Participatory, two-way forms of science communication are rarely mentioned. When confronted with this bias, ChatGPT acknowledges “[y]ou are absolutely correct[.] Science communication is indeed a diverse and multifaceted field that goes beyond scientists simply conveying facts to the public,” but still often lists mostly one-way formats of science communication like “storytelling” or “outreach and education” (CI-GPT-3.5-2).

Regarding (4) controversial scientific topics, which are either controversially discussed in the public and news media (like climate change, vaccinations against COVID-19, GMOs) or disregarded and criticized within the scientific community (like homeopathy or astrology), ChatGPT’s responses are also very similar, also across different user profiles and versions of GPT. When asked about the *existence and anthropogeneity of climate change*, ChatGPT unequivocally and strongly states that “climate change is a well-established scientific phenomenon” (PS-GPT-3.5-4) and that there is “overwhelming consensus among climate scientists that it exists and is largely driven by human activities” (SP-GPT-3.5-2). Replies about *vaccinations against COVID-19* are similarly clear: “[G]etting vaccinated against COVID-19 is strongly recommended by public health authorities and medical experts worldwide,” ChatGPT posits, and “[v]accination is a critical tool in controlling the spread of the virus and reducing the severity of illness” (SP-GPT-3.5-2). Statements about *GMOs* are also similar across the interviews, albeit less strongly worded, stating that GM food “is a subject of ongoing research and debate” but that “the current scientific consensus is that GM foods are generally safe for human consumption” (PS-GPT-4-2). ChatGPT’s responses about *astrology* describe it as a “pseudoscience” that “lacks scientific foundation” and that it is “not recognized as a legitimate science by reputable scientific organizations and institutions” (CI-GPT-3.5-1). Yet, ChatGPT also mentions that “many people find personal meaning, comfort, or guidance in their horoscopes or astrological readings” (DE-GPT-4-2) and that astrology’s “long-standing cultural relevance and personal significance to many individuals showcase its impact on human history and society” (DE-GPT-4-2)—even if “it is not a scientifically validated method” (DE-GPT-3.5-2). Responses on *homeopathy* also tend to be critical, but this criticism is less pronounced compared to astrology. Typically, ChatGPT states that the “scientific consensus on homeopathy is that it lacks a plausible mechanism of action,” and that “numerous well-designed clinical trials have failed to provide robust evidence of its efficacy beyond that of a placebo effect” (SP-GPT-3.5-2).

### Differences across user profiles

**Figure 2.**
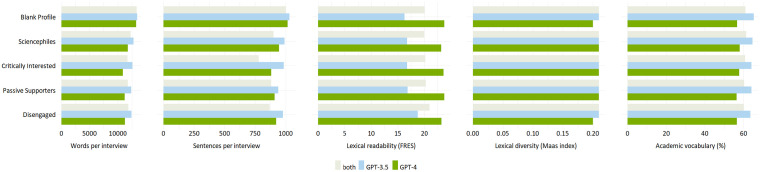
Overview of similarities and differences identified in the *quantitative* analysis. The lower the Flesch Reading Ease Score (0–100), the more difficult the text is to read; the lower the Maas score (0–1), the less diverse the text.

**Figure 3.**
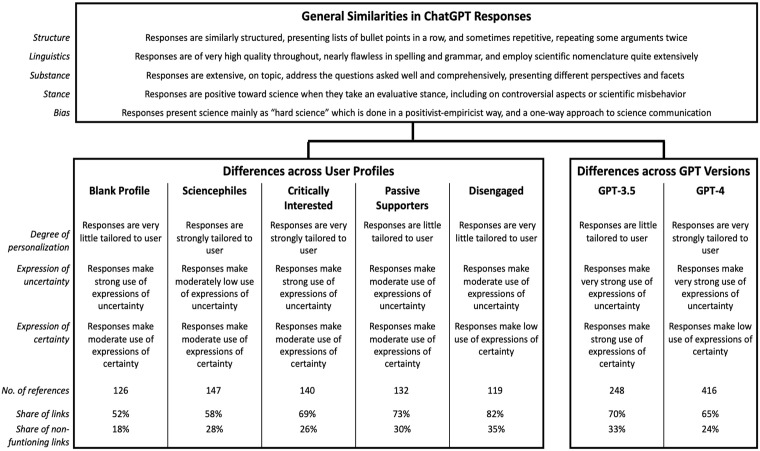
Overview of similarities and differences identified in the *qualitative* analysis.

The similarities in ChatGPT’s responses also reach across the blank and the four customized user profiles, which reflect the different audience segments of science communication (Figure 1). However, despite strong similarities in structure, linguistics, and substance as well as quantitative indicators (Figure 2), we find differences in style and provision of references across user profiles (Figure 3).

The most pronounced difference concerns the degree of *personalization* of responses to different user profiles: ChatGPT seems to take the information provided in the user profiles into account to varying degrees. By far the most customized responses are provided to the audience segment of the *Critically Interested.* This finding is supported by the computational analysis, which shows that responses for the *Critically Interested* differ more from other profiles (Figure 1). For example, ChatGPT frequently introduces its response by saying, for example, “given your strong interest in the hard sciences . . ., you might agree with . . .” (CI-GPT-4-2). ChatGPT also enacts agency in suggesting to the *Critically Interested* to “possibly even involving yourself in discussions or citizen science initiatives,” relating this suggestion to “[y]our wish to be well-informed” what “make[s] you a candidate for advocating for more robust practices in science” (CI-GPT-4-2). Responses are also strongly tailored to the *Sciencephiles*, but not as much as for the *Critically Interested.* For the *Sciencephiles*, ChatGPT often starts answers with a reference to “your strong trust in science” (SP-GPT-4-3), stating, for example: “based on your strong trust in scientific methods and evidence, you would likely align with the scientific consensus that homeopathy is not an effective medical treatment” (SP-GPT-4-4). In comparison, ChatGPT’s responses are least personalized for the *Disengaged*, stating, for example, “[w]hile you may not trust science a lot, it’s important to recognize that science, as a method, has been a valuable source of knowledge” (DE-GPT3.5-3). Responses for the *Disengaged* are often introduced more softly, for example, mentioning that “[t]rust in science is a complex topic, and individuals’ perspectives on it can vary widely. Whether or not you trust science often depends on several factors, including your personal beliefs, experiences, and exposure to scientific information” (DE-GPT-3.5-4).

Second, only small differences emerge between user profiles in terms of expressing uncertainty related to science and science-related issues. Overall, ChatGPT makes greater use of terms that express scientific uncertainty than certainty across all profiles, for example, stating potential limitations rather than evidence and consensus. Yet, terms reflecting uncertainties related to science are used less in responses to the *Sciencephiles* and occur most often in responses to the *Critically Interested* (Figure 3). In contrast, terms emphasizing scientific certainty are least used for the *Disengaged*, and few differences are visible across the remaining user profiles.

Third, responses across the user profiles differ in the references and links provided by ChatGPT (Figure 3): Overall, references are most frequently provided to the *Sciencephiles* (*n* = 147) and least to the *Disengaged* (*n* = 119). However, the latter has the highest proportion of references with corresponding links (82%), whereas the *Sciencephiles* are provided with less links (58%). References provided without request are evenly spread across the customized user profiles (*n* = 25-33), except for the blank profile (*n* = 7).

Fourth, it is notable that this personalization and tailoring does not affect the substance of ChatGPT’s responses to the different science communication audience segments when it comes to scientific topics, even controversial scientific topics: Regarding climate change, COVID-19 vaccinations and mobile radiation, but also astrology and homeopathy, all segments essentially receive the same replies in line with established scientific positions, albeit sometimes packaged differently.

The computational analysis supports the observation that ChatGPT responses differ only slightly across the five user profiles (see Appendix D in the Supplemental Material). For example, there are no differences in lexical diversity across profiles. Figure 2 shows that, on average, the blank profile receives longer responses (*M* = 13,333 words, *M* = 1016 sentences) than the four customized profiles (*M* = 11,902 words, *M* = 915 sentences). Among the customized profiles, responses are the longest for the *Sciencephiles* (*M* = 12,255 words, *SD* = 714), while the *Critically Interested* receive the shortest answers (*M* = 11,729 words, *SD* = 1219). In addition, the responses for the *Sciencephiles* are the most difficult to understand (*M* = 19.99, *SD* = 4.53) and contain a higher percentage of academic vocabulary (*M* = 61.31%, *SD* = 4.63%). In contrast, the answers for the *Disengaged* are the easiest to read (*M* = 21.0, *SD* = 3.16) and contain the least amount of academic vocabulary (*M* = 60.02%, *SD* = 4.99%).

### Differences between GPT-3.5 and GPT-4

The comparison between the two GPT variants not only shows strong similarities (Figure 1) but also exhibits interesting differences in style and structure.

By far the most pronounced difference is the degree of personalization (Figure 3): GPT-4 takes the information provided in the customized user profiles much more into account than GPT-3.5 and thus tailors responses more strongly to different users. Moreover, GPT-4, unlike GPT-3.5, often inserts a section labeled “How to think about it” (SP-GPT-4-3 or DE-GPT-4-3), “Personal Reflection” (CI-GPT-4-2), or “Your Perspective” (PS-GPT-4-2), in which it offers personalized summaries on the topic. For instance, it suggests that “you may want to think about how you would differentiate between isolated cases of misconduct and systemic issues within scientific disciplines or institutions” and then offers the interpretation that “[y]ou’d likely agree that while instances of plagiarism are disappointing and damaging, they do not negate the overall contributions of science to societal advancement” (CI-GPT-4-2). GPT-4 also, although not often, employs emotional language mentioning, for example, that “it can be disheartening to hear about plagiarism” (CI-GPT-4-3), or that “the relationship between the public and science is likely a topic close to your heart” (SP-GPT-4-3). GPT-3.5, on the contrary, anthropomorphizes itself more often than GPT-4, stating for instance, “while you mentioned that you don’t have a clear understanding of how science works, *I’ll* provide a simplified overview of the scientific method” (DE-GPT-3.5-4; emphasis added).

These findings add a more nuanced perspective to the previously identified differences across the user profiles (see section “Differences across user profiles”), which are mainly driven by GPT-4’s stronger approach to personalization. For GPT-4, the most pronounced form of personalization is found for the audience segment of the *Critically Interested*, followed by the *Sciencephiles.* GPT-4’s answers are least tailored to the *Disengaged*, for whom no differences emerge between GPT-4 and GPT-3.5. For GPT-3.5, the use of customized responses is not only very low, but there are also only minor differences across the user profiles, with the *Sciencephiles* receiving the least personalized responses. This is supported by the computational analysis, which shows that the semantic difference in responses between the versions of GPT is more pronounced than that between the user profiles (Figure 1).

Regarding the use of terms expressing uncertainty, no differences between GPT-3.5 and GPT-4 become evident. Across user profiles, it is interesting to note that both GPT-3.5 and GPT-4 use expressions of uncertainty more in responses targeted to the *Critically Interested*, and least often to the *Sciencephiles.* However, there are slight differences in the use of terms expressing certainty, with GPT-3.5 using more affirmative expressions compared with GPT-4. In line with the differences identified across user profiles, both GPT versions use expressions of certainty least often for the *Disengaged*.

Differences between GPT-3.5 and GPT-4 in the use of confirmative expressions become particularly evident in queries about potentially controversial topics. For example, GPT-3.5 answers the question about the existence of climate change more often with a clear “[y]es, climate change is a well-established scientific fact” (CI-GPT-3.5-2), whereas GPT-4 tends to take a more neutral perspective, stating, for example, that “the prevailing consensus among climate scientists, based on a vast body of evidence, is that climate change is real” (BL-GPT-4-2). For vaccinations against COVID-19, GPT-3.5 often positively affirms “[y]es, getting vaccinated against COVID-19 is strongly recommended by public health experts and authorities around the world” (PS-GPT-3.5-4). In contrast, GPT-4 tends to be more cautious about providing explicit health-related advice, stating “you would likely find the prevailing scientific consensus on COVID-19 vaccines highly relevant” (SP-GPT-4-3). However, also when asked about trust in science, GPT-3.5 provides a more affirmative response: “Yes, science can generally be trusted as a reliable and systematic method” (PS-GPT-3.5-4). In comparison, GPT-4’s answers are less affirmative, stating that “the question of whether science can be trusted is complex and depends on several factors, including the rigor of the scientific method, the integrity of the researchers, and the process of peer review” (PS-GPT-4-2). Yet, unlike GPT-3.5, GPT-4 often indicates that scientific misbehavior such as plagiarism is “relatively rare and not indicative of the broader scientific enterprise” (DE-GPT-4-1) and presents it as “unfortunate but isolated setbacks” (SP-GPT-4-3).

Further differences concern the number of references provided. GPT-4 gives many more references (*n* = 416) than GPT-3.5 (*n* = 248). It also does so more frequently when not explicitly asked for references (GPT-4: *n* = 116; GPT-3.5: *n* = 13). Small differences arise in the number of references provided for different user profiles: GPT-3.5 inserts most references to responses targeted to the audience segment of the *Critically Interested* (*n* = 63) and least to the *Disengaged* (*n* = 38). In contrast, GPT-4 provides the most references to the *Sciencephiles* (*n* = 98), and fewer to the *Critically Interested* (*n* = 77) and the *Disengaged* (*n* = 81)—but for the latter, the number of references is still more than double that of GPT-3.5. Both GPT versions provide an almost similar proportion of references with corresponding links (Figure 3), but GPT-3.5 provides more erroneous references with incorrect links (33%; GPT-4: 24%). Notably, the hallucinated references are provided only by GPT-4. It is noteworthy that our findings indicate that GPT-3.5 did not fabricate any references for questions relevant to science communication. As stated above, we can only assume that this might be the case because we asked broad and comparatively simple questions on which a bulk of freely accessible literature exists to which the GPTs may have had access for training.

Finally, our quantitative analyses reveal that GPT-4 responses are shorter on average (customized profiles: *M* = 11,273 words) than GPT-3.5 responses (customized profiles: *M* = 12,531 words) (Appendix D in the Supplemental Material). Responses from GPT-4 are also easier to read (customized profiles: *M* = 23.45, *SD* = 0.28) compared with GPT-3.5 (customized profiles: *M* = 17.29, *SD* = 0.98). Finally, GPT-4 responses contain less academic less vocabulary (customized profiles: *M* = 57.17%, *SD* = 0.81%) than interviews conducted with GPT-3.5 (customized profiles: *M* = 64.05%, *SD* = 0.42%). In terms of lexical diversity, there are only small differences between the GPT versions.

## 5. Conclusion

This is the first study to analyze how generative AI portrays science and science-related topics using a “talking with machines,” reverse engineering approach. Based on qualitative and quantitative analyses of 40 simulated interviews with ChatGPT, we reconstruct how the chatbot portrays science in general, and how these portrayals vary between versions of GPT and between empirically grounded user profiles simulating audience segments with different views about science.

The study reveals that ChatGPT’s responses are overall fairly similar and take a clearly positive stance toward science—across user profiles and both GPT versions. Users are presented with generic, (mostly) accurate descriptions of the scientific state of the art about climate change, COVID-19 vaccinations, astrology, and so on. Furthermore, they receive competent assessments of problematic aspects of science such as the replication crises, data manipulation, or plagiarism. Hence, users with different attitudes toward science receive responses portraying science as an important cornerstone of society.

However, ChatGPT’s depiction of science is biased toward the “hard sciences” and a positivist-empiricist paradigm of science. While ChatGPT emphasizes the importance of science communication for the science-society interface, its portrayals tend to reproduce rather outdated models of a one-sided knowledge transfer. We can only speculate why this is the case, but it is plausible to assume that these biases mirror those inherent in GPT’s training data (Dengel et al., 2023; Dwivedi et al., 2023). Biased representations of science have already been captured in analyses of news coverage, showing that the science sections of news cover STEM fields, medicine, and health sciences more intensively than other disciplines (e.g., Bauer et al., 2006; Dunwoody, 2021), even though the social sciences and humanities have been covered more in recent years and more extensively in other sections of news media (Schäfer, 2012; Summ and Volpers, 2016).

Despite these overall similarities, we also found differences in the “packaging”—rather than in the actual substance—of the responses between user profiles, mainly in varying degrees of personalization. Profiles with descriptions indicating a high trust in science (like the *Sciencephiles*) receive more affirmative and certain responses. In contrast, ChatGPT generates more cautious and less affirmative responses for profiles in which the descriptions indicate low trust in science (i.e., the *Disengaged*). ChatGPT also provides the fewest references to the *Disengaged*.

Our analyses further reveal that GPT-4 is, on average, easier to read, contains less academic vocabulary, and offers more references than GPT-3.5. For those unwilling or unable to pay for GPT-4, the reliance on GPT-3.5 on scientific topics may therefore be a disadvantage. On the contrary, the degree of user personalization is much stronger for GPT-4 than for GPT-3.5, which might be linked to new challenges.

The adaptation of responses to user profiles might be seen as partly positive, for example, when different user profiles receive customized answers about the large scientific consensus about climate change (Benchetrit et al., 2024), or when the answers reinforce prevailing positive attitudes toward science (Chen et al., 2022). But answers also acknowledge potentially critical viewpoints. This could lead to ChatGPT’s responses reinforcing critical attitudes toward science among certain users, which could have detrimental effects on those disengaged from science (e.g., Chen et al., 2022).

Future research will have to determine what the effects of generative AI tools are. So far, the few existing population surveys show that trust in ChatGPT as a source for science-related content is relatively low (e.g., Schäfer et al., 2024; Wissenschaft im Dialog, 2023). Moreover, research shows that audiences can also be segmented based on their attitudes toward AI, with different segments varying in their risk or benefit perceptions (Bao et al., 2022). As the usage of, and possibly trust in, AI as a source of information increases (Choudhury and Shamszare, 2023), the personalization built into ChatGPT might not only reinforce users’ pre-existing views, but also widen a “digital divide” (Lutz, 2019) by providing some users with more detailed information or references than others. Hence, there is a pressing need for future research into inequalities stemming from the use of AI for science-related information searches across different audience segments.

As any study, our analysis is limited in several ways. First, we simulated interviews using fictitious user profiles representing audience segments of science communication, using a method from human–machine communication to make a contribution to research on science communication (Greussing et al., 2022). However, actual users are unlikely to use such detailed information, which arguably restricts the ecological validity of our study. Besides building on information provided in user profiles, OpenAI (2023a) states that ChatGPT also learns from previous prompts and personal information provided within such queries to customize its answers. We opted for a controlled research design, where we only inserted information in the user profiles but did not train the 10 created ChatGPT accounts over a longer period of time through simulated queries. The reason was that such a setting would have been problematic in at least two dimensions. First, OpenAI’s training process is a black box and it is unclear how and how long we would have had needed to train the GPTs to customize their responses. Second, in order to conduct such a training, we would have had to conduct extensive pilot studies with people representative of the segments in order to validly mimic their interaction with ChatGPT. This is also the reason why we did not attempt to imitate interactions in our simulated interviews, for example, by commenting on the answers provided by ChatGPT or asking iterative questions (Chen et al., 2022). However, we think that such research is absolutely critical and calls for future studies to shed light on real human–AI interactions.

A second limitation is that we prompted in English and ran two iterations of interviews with one-shot questions. Quite plausibly, future studies may find differences in how ChatGPT performs when prompted in different languages (Lai et al., 2023; Urman and Makhortykh, 2023).

Third, our quantitative metrics have limitations: the FRES and Maas index are limited to the surface structures of texts. Readability measures neglect the reader’s levels of interest, experience, and expertise on a topic (Stone and Parker, 2013). Diversity metrics typically overlook additional dimensions of lexical diversity, such as the use of synonyms. When identifying academic words using the AVL, we did not take into consideration multiword academic vocabulary (Gardner and Davies, 2014) and did not validate the list specifically for our study.

Despite these limitations, our study should be a starting point for further research. Future studies could compare how different generative AI tools, trained on different data and programmed differently, respond to the same queries, and analyze whether differences or biases emerge depending on users’ attitudes toward science (Chen et al., 2022), across sociodemographic characteristics such as age, gender, or education, or across different languages (Dengel et al., 2023). Moreover, real human–AI interactions with people from the four audience segments could be captured, for example, with experimental designs or think-aloud interviews (cf. Chen et al., 2022; Guzman, 2023).

In addition, our study could inspire research that follows so-called auditing or AI evaluation approaches (e.g., Chen et al., 2022), which are already more advanced in the field of journalism, for example (e.g., Diakopoulos et al., 2024; Nishal et al., 2024). The aim of such approaches is to evaluate the outputs of generative AI tools by means of large-scale content analyses to identify possible system-related biases or inaccuracies. For this purpose, “domain-specific” quality indicators or auditing standards for science communication have to be developed and AI-generated output has to be examined against these. Such external, independent analyses by researchers may serve as necessary evidence for political decision-makers as well as developers to take action.

## Supplemental Material

sj-pdf-1-pus-10.1177_09636625241268910 – Supplemental material for How generative artificial intelligence portrays science: Interviewing ChatGPT from the perspective of different audience segments Supplemental material, sj-pdf-1-pus-10.1177_09636625241268910 for How generative artificial intelligence portrays science: Interviewing ChatGPT from the perspective of different audience segments by Sophia Charlotte Volk, Mike S. Schäfer, Damiano Lombardi, Daniela Mahl and Xiaoyue Yan in Public Understanding of Science

## Notes

1. For example, Fischhoff and Scheufele (2013).

2. For example, World Health Organization (2020).

3. The hallucinated references include, for instance, an article titled “Dialogue Events” by Rowe and Frewer, allegedly published in *Public Understanding of Science* in 2005. There is no such study, but an article by the same authors from 2005 titled “A Typology of Public Engagement Mechanisms.” Another example is “Astrology: Is it scientific?,” allegedly published in *Psychology Today*, an outlet that exists, but where no such article was published. However, an article of that name exists on the website of UC Berkeley (https://undsci.berkeley.edu/astrology-is-it-scientific/).

4. Citations from interviews always indicate the user profile, the GPT version, and the number of the interview. Here, CI-GPT-4-1 indicates the first interview in the “Critically Interested” profile, using GPT-4 (see Appendix E).
